# Primary Syphilis of the Oral Cavity, a Rare Presentation of a Re-Emerging Disease

**DOI:** 10.7759/cureus.14595

**Published:** 2021-04-20

**Authors:** Mohammed Bindakhil, Faizan Alawi, Katherine France, Takako I Tanaka

**Affiliations:** 1 Oral Biology and Diagnostic Sciences, Dental College of Georgia, Augusta University, Augusta, USA; 2 Division of Dermatopathology, Department of Dermatology, Hospital of the University of Pennsylvania, Philadelphia, USA; 3 Division of Oral Medicine, Hospital of the University of Pennsylvania, Philadelphia, USA

**Keywords:** bacterial sexually transmitted infections, oral diseases, oral infectious diseases, chronic ulcer

## Abstract

Syphilis is a sexually transmitted disease caused by microaerophilic spirochete *Treponema pallidum*. After contact, *T. pallidum* infiltrates the genital or oral mucosa and spreads systemically throughout the blood and lymphatic systems. Syphilis is classified into stages, with varying signs and symptoms associated with each stage. Primary syphilis has an incubation period of up to 90 days following the sexual transmission of *T. pallidum*, and is characterized by the development of chancres at the site of inoculation. Rarely, primary syphilis presents in the tongue, which can mimic many other conditions that affect the oral cavity. In this article, we discuss a rare oral manifestation of primary syphilis.

## Introduction

Syphilis, a sexually transmitted disease caused by the microaerophilic spirochete bacterium *Treponema pallidum*, is considered a global public health issue [1]. This bacterial pathogen was first discovered in Europe toward the end of the 15th century [1]. Thus far, the highest reported frequency of syphilis cases was in the 1940s, which was initially attributed to World War II [1]. With the advent of antibiotics, incidence of this disease reduced drastically as compared to the 16th century [1,2]. However, since 2000, reported cases of syphilis in the USA have tripled [1,2]. Although most of the reported cases of syphilis occurred in men who have sex with men, the heterosexual transmission of the disease cannot be overlooked [1].

Following sexual contact with an infected person, *T. pallidum* penetrates the genital or oral mucosa and spreads throughout the body via the blood and lymphatic systems [1]. Early detection and treatment can significantly decrease the complications associated with the disease. Although uncommon, primary syphilis should be considered in the differential diagnosis of non-healing oral ulcerations. Here, we present an unusual oral manifestation of primary syphilis. This case was previously presented as a ‘’text-only’’ virtual poster at the 2019 annual meeting of the American Academy of Oral and Maxillofacial Pathology on June 7, 2019.

## Case presentation

**Figure 1:**
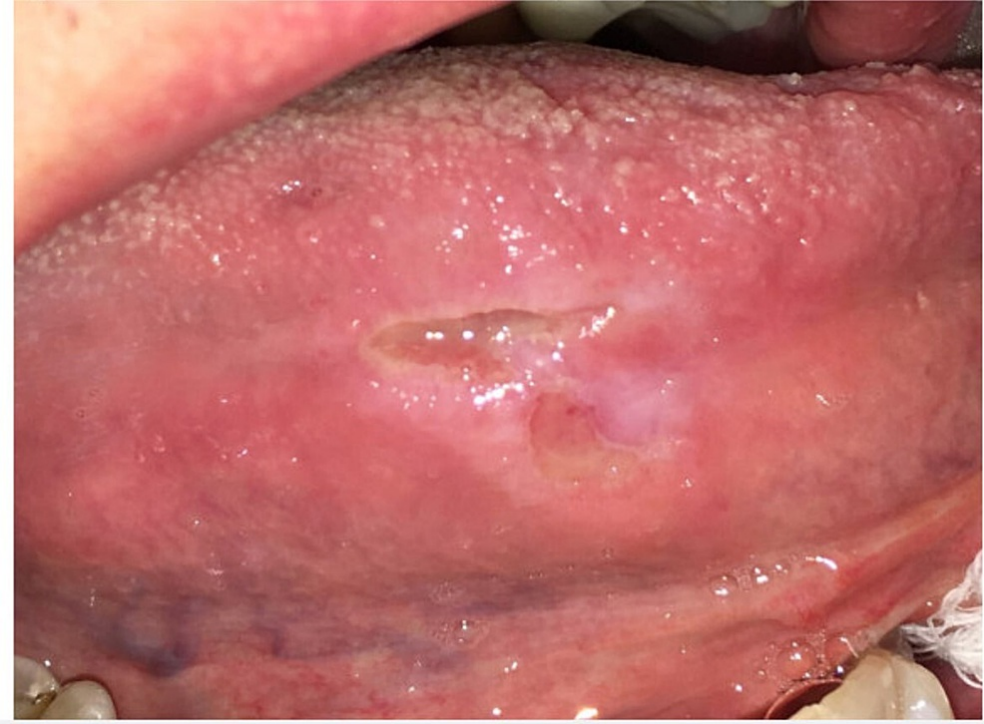
Tongue ulceration measuring about 1 cm on the right lateral tongue surrounded by lichenoid-appearing inflammation

A 52-year-old man presented with a non-healing tongue ulcer and erythema, which had been persisting for one month. The patient reported an occasional burning sensation and discomfort but did not indicate severe pain. He defined himself as a homosexual male who had always enjoyed good health and had not been placed on routine medications, nor had he abused any drugs. He denied fever, weight loss, and neurological symptoms. Extraoral examination was unremarkable without evidence of lymphadenopathy. Intraoral examination showed deep, non-indurated, ulceration of about 1 cm located on the right side of the tongue and encircled by mixed white and erythematous inflamed borders (Figure 1). 

**Figure 2:**
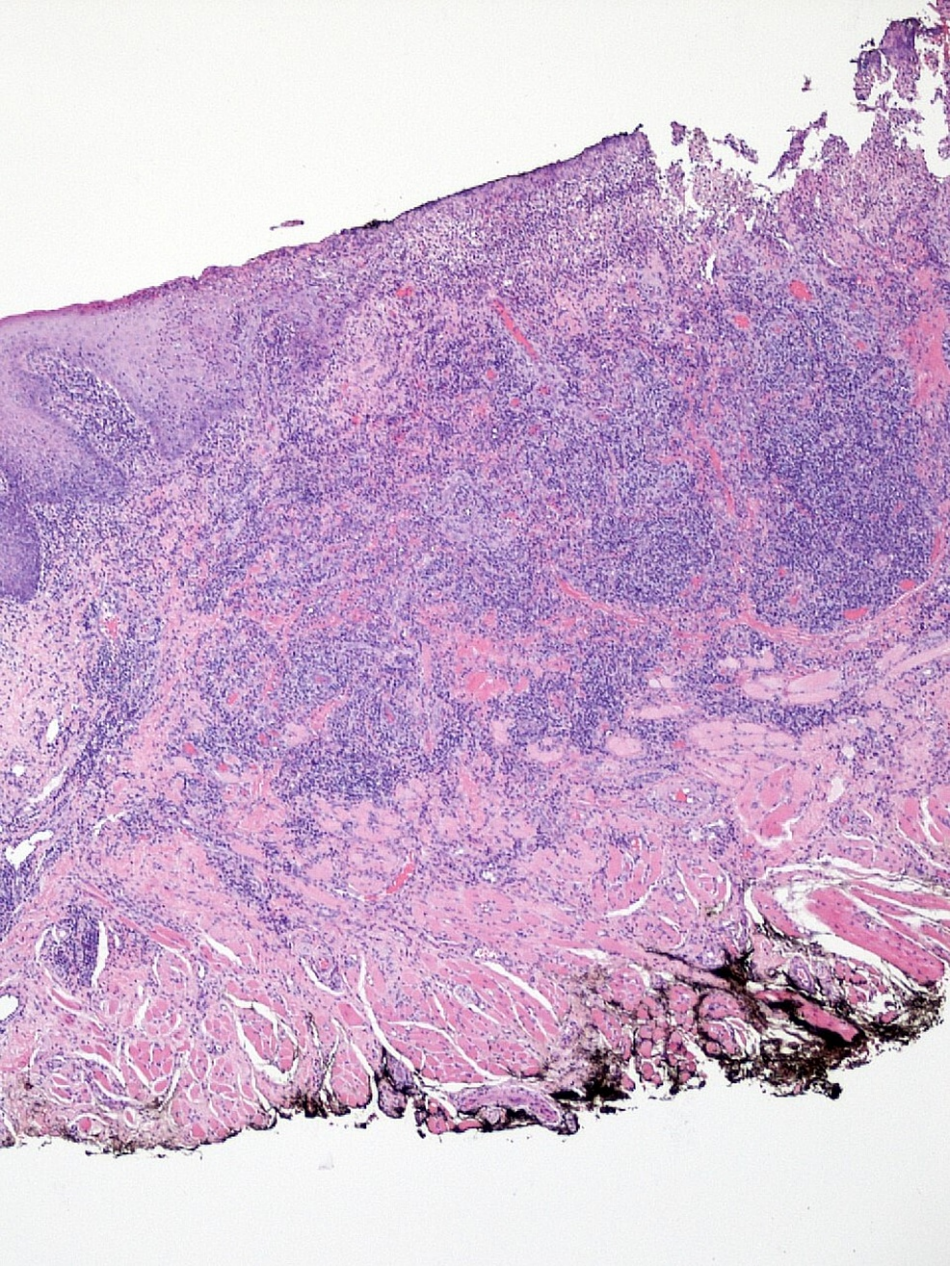
: Mucosal fragment exhibiting ulcerated mucosa overlying lamina propria containing a dense, deep, and perivascular lymphoplasmacytic inflammatory infiltrate Hematoxylin and eosin, magnification x100

Based on the clinical examination, an excisional biopsy of the ulcerated lesion was recommended. The differential diagnosis included traumatic, neoplastic, and infectious etiologies of ulceration. Incisional biopsy from the tongue lesion revealed ulcerated epithelium overlying connective tissue containing deep lymphoplasmacytic inflammatory infiltrate located around the blood vessels (Figure 2). 

**Figure 3:**
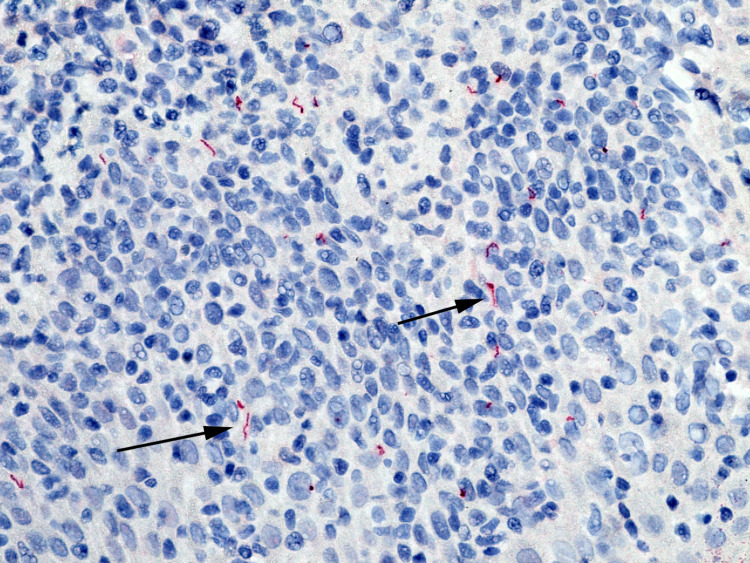
A Treponema pallidum immunohistochemical stain revealed spirochetal organisms (black arrows)

Periodic acid-Schiff stain tested negative for fungi. However, immunohistochemical studies for *T. pallidum* revealed a number of spirochetal organisms (Figure 3).

Serology demonstrated the presence of rapid plasma reagent (RPR) titers and *T. pallidum* antibodies. The overall findings confirmed that the patient had primary syphilitic chancre. After learning about the diagnosis, the patient reported engaging in sexual intercourse that involved oral contact within the six weeks prior to the development of the tongue ulceration. The patient was treated with a single-dose intramuscular injection of benzathine penicillin G (BPG) at 2.4 million units (MU). Three weeks later, the patient presented for a follow-up visit, by which time the ulceration had resolved.

## Discussion

The different stages of syphilis are characterized by a number of notable signs and symptoms [1-4]. Primary syphilis is the first stage, with an incubation period of about 90 days after sexual contraction of *T. pallidum* [1-4]. It is characterized by the formation of chancres at the area of inoculation. Typically, these chancres are painless, well-encircled, indurated ulcers characterized by raised borders [1-4]. Chancres can vary significantly in clinical manifestation [1]. This variation, in addition to the painless nature of the lesions, may explain why about two-fifths of patients are not diagnosed at the primary stage. Spontaneous healing of chancres occurs within six weeks without leaving a scar [1]. If not treated within eight weeks, primary syphilis advances to secondary syphilis as the bacterial infection spreads through the blood [1].

Secondary syphilis is characterized by the intravascular spreading of *T. pallidum* from the site of infection, and occurs in no fewer than 25% of patients [1,2,4,5]. The characteristics of secondary syphilis include cutaneous eruptions on the soles and palms, fever, genital condyloma latum, lymphadenopathy, and sore throat [1,6]. In 30-40% of secondary syphilis patients, the mucosa is involved, manifesting in the form of mucous patches, painful ulcerations, and painless maculopapular lesions. As with the primary stage, secondary syphilis is also contagious [2-7].

About 30% of syphilis patients eventually progress to tertiary syphilis [1]. Tertiary syphilis has debilitating effects on the functions of several body systems [1,7]. Usually at this stage, there are late clinical manifestations that appear 15-30 years following the initial infection [4,8]. Among these are inflammatory diseases of the skin, such as gummatous lesions; diseases of the cardiovascular system, such as coronary vessel disease and aortitis; and diseases of the bone, such as osteitis [4,8]. Occasionally, late clinical manifestations involve the reproductive organs, eyes, oral cavity, respiratory tract, skeletal muscle, abdominal organs, and lymph nodes [4,8]. In the latent stage--that is, the period between the secondary and tertiary stages of infection--clinical symptoms may disappear while serology is still reactive [1].

The extragenital lesions in primary syphilis develop in no more than 14% of affected patients [6-9].The manifestations of syphilis in the oral cavity often occur at the secondary stage, while rarely occurring in the primary and late stages [4,6]. When the oral cavity is affected at the primary stage, it is usually following oro-genital contact [6-9]. While oral chancres usually develop on the lips, they may occasionally occur on the tongue and palate, and sometimes manifest as patches on the mucous membrane [1]. While in this case the primary syphilis chancre involved the lateral border of the tongue, it may occur on the tip of the tongue as in the case observed by Fregnani ER et al. [10].

Patches typically appear as oval-shaped, well-demarcated erythematous or thin white plaques on an erythematous base; these patches may affect the lips, buccal mucosa, and tongue [1,6]. There may also be an appearance of papular erythematous plaques (plaques faucheés), aphthous-like superficial ulcers, or irregular serpiginous erosions (snail-track ulcers) [1,6]. At the commissures, fissured papular lesions or split papules may also be present [1].

Typically, the diagnosis of syphilis is achieved through serologic testing utilizing one of two algorithms [1]. The standard screening algorithm starts with a non-treponemal blood test, a venereal disease research laboratory test or RPR, and finally a treponemal test (e.g., an automated enzyme or chemiluminescence immunoassay or *T. pallidum* particle-agglutination test) [1]. At the microscopic level, the perivascular infiltration of lymphocytes, plasma cells, and histiocytes can be seen irrespective of the stage [1]. Treponemes are often found in primary and secondary lesions, and can be detected using either the Warthin-Starry stain or immunohistochemical staining [5].

Over time, BPG has been regarded as the drug of choice for treating syphilis at all stages of infection [5]. The recommended dose for the treatment of early and uncomplicated stages of adult infection is 2.4 MU, in a single intramuscular injection [1]. It is necessary to repeat this treatment once every week for two more weeks in cases of tertiary or late latent stage syphilis, or in cases with an unknown duration of infection [1].

After the treatment is complete, a confirmatory serologic cure is rendered when a reduction in treponemal antibody titers by a factor of >4 is observed within 6-12 months or 12-24 months following the therapy for early syphilis and late syphilis, respectively [7].

## Conclusions

The clinical oral manifestations of primary syphilis are similar to those of many other diseases affecting the oral cavity. If syphilis is detected and treated at an early stage, the complications emanating from the disease will be significantly reduced. In the differential diagnosis of non-healing oral ulcerations, primary syphilis should be considered regardless of the rarity of occurrence.
